# Analysis of the epidemiological status, microbiology, treatment methods and financial burden of hematogenous osteomyelitis based on 259 patients in Northwest China

**DOI:** 10.3389/fendo.2022.1097147

**Published:** 2023-01-04

**Authors:** Shuaikun Lu, Linhu Wang, Wen Luo, Guoliang Wang, Zhenfeng Zhu, Yunyan Liu, Hao Gao, Congxiao Fu, Jun Ren, Yunfei Zhang, Yong Zhang

**Affiliations:** ^1^ Department of Orthopaedics, Tangdu Hospital, Air Force Medical University, Xi’an, China; ^2^ Department of Ultrasound, Xijing Hospital, Air Force Medical University, Xi’an, China

**Keywords:** calcium sulfate/calcium phosphate bone cement, epidemiological, financial burden, hematogenous osteomyelitis, *Staphylococcus aureus*

## Abstract

**Background:**

The incidence of hematogenous osteomyelitis is on the rise, and the prognosis is poor. There has been no large-scale epidemiological analysis of hematogenous osteomyelitis in the world, and the treatment method is still controversial.

**Methods:**

A retrospective case study method was used to collect and analyze clinical data obtained from patients with hematogenous osteomyelitis in a tertiary hospital in Northwest China from January 1, 2011, to December 31, 2020. The aim of this study was to investigate the epidemiological status, microbiological characteristics, treatment and financial burden of hematogenous osteomyelitis in Northwest China to explore the therapeutic effects of different treatment methods, elucidate the epidemiological characteristics of hematogenous osteomyelitis and to provide a basis for the choice of treatment.

**Results:**

We included 259 patients with hematogenous osteomyelitis, including 96 patients with acute hematogenous osteomyelitis and 163 patients with chronic hematogenous osteomyelitis. The cause of the disease was not obvious in most patients, the sex ratio of males to females was 1.98, and the three most common infected sites were the tibia, femur and phalanx. Regarding preoperative serum inflammatory markers, the rate of positivity for ESR was the highest at 67.58%. Among pathogenic microorganisms, Staphylococcus aureus was the most common. Regarding the financial burden, the median total cost per patient was 25,754 RMB, and medications accounted for the largest proportion of the main costs.

**Conclusions:**

The most common pathogen associated with HO infection was MSSA. Oxacillin has good PK and PD and is recommended as the first-line drug. Some blood-borne bone infections may lead to complications, such as pulmonary infection through bacteremia, which requires early detection to avoid a missed diagnosis. Regarding surgical intervention, debridement plus absorbable calcium sulfate bone cement and calcium sulfate calcium phosphate bone cement exclusion have achieved good therapeutic effects, but they are worthy of further in-depth research. Regarding the financial burden, the median total cost per patient was 25,754 RMB. The financial burden of blood-borne osteomyelitis was lower than that of traumatic osteomyelitis. Among the main costs, drugs accounted for the largest proportion.

## 1 Introduction

Osteomyelitis is an inflammatory bone disease caused by microbial infection that leads to bone destruction. It is mainly divided into hematogenous osteomyelitis (HO), traumatic osteomyelitis and diabetic foot osteomyelitis (1, 2). According to the course of the disease, it is mainly divided into acute osteomyelitis and chronic osteomyelitis, and the clinical duration of acute and chronic osteomyelitis ranges from several weeks to several months (2–5).

Hematogenous osteomyelitis is caused by the accumulation of bacteria in the bloodstream and in tissues, and extensive proliferation leads to bone infection, which can be induced by local soft tissue sprains, contusions, stab wounds, or skin damage/infection and mainly involves the metaphysis of long bones.

The aim of this study was to investigate the epidemiological status, microbiological characteristics, treatment and financial burden of hematogenous osteomyelitis in Northwest China to explore the therapeutic effects of different treatment methods, elucidate the epidemiological characteristics of hematogenous osteomyelitis and to provide a basis for the choice of treatment.

Regarding the epidemiology of osteomyelitis worldwide, the incidence of osteomyelitis in the United States doubled from 11.4 cases per 100,000 people between 1969 and 1979 to 24.4 cases per 100,000 people between 2000 and 2009 (6). The incidence of osteomyelitis, excluding vertebral osteomyelitis, in Germany has increased from 15.5 cases per 100,000 in 2008 to 16.7 per 100,000 in 2018, which is an increase of 10.44% (7). A study of bacteremic bone and joint infections in Spain (1982-2011) showed that in Spain, the incidence of bacteremia-related bone and joint infections increased from 2.34 cases per 100,000 people per year to 5.78 cases per 100,000 people, with the number of osteomyelitis cases also increasing (8). A survey of children with acute hematogenous osteomyelitis (AHO) in Israel showed that the incidence of acute hematogenous osteomyelitis was 5.16 cases per 100,000 people from 2005 to 2012; the incidence of AHO in Bedouin and Jewish individuals was 7.3 per 100,000 and 4.1 per 100,000, respectively (9). A study of osteomyelitis in Uganda revealed a 10% prevalence of osteomyelitis in orthopedic clinics (10). In Asia, a nationwide study of childhood bone and joint infections (2008-2016) in Korea revealed that the incidence of osteomyelitis was between 7.8 and 9.1 cases per 100,000 people per year (11). Not only is the incidence increasing daily, but the cost of treatment is also a troubling problem. According to a French study of bone and joint infections in 2008, 0.2% of all hospitalized patients in France exhibited bone and joint infections, accounting for 54.6 cases per 100,000 people. The average treatment cost per case of osteomyelitis is 5,422 euros (12). There are reports on the financial burden of traumatic osteomyelitis in China, and the median treatment cost is 73,528 RMB, of which the material cost accounts for the largest proportion at 61% of the total cost (13). The financial burden of hematogenous osteomyelitis has not been reported in the literature.

Up to now, there is a lack of large-scale epidemiological research on hematogenous osteomyelitis worldwide. China still lacks national epidemiological data on hematogenous osteomyelitis. Jiang (14) et al. provided epidemiological data on chronic osteomyelitis of the extremities in Southern China from 2010 to 2015, and Wang (15) et al. provided epidemiological data related to chronic osteomyelitis in Southwest China in 2016. Ma (16) et al. provided epidemiological data related to chronic osteomyelitis in Northern China from 2007 to 2014. These data are mainly derived from investigations of traumatic osteomyelitis and include some cases of chronic hematogenous osteomyelitis (CHO) and can be used by government departments and medical departments for reference and use. Importantly, there is a lack of relevant analysis on the treatment strategy for hematogenous osteomyelitis.

There is currently a lack of data and related studies on the epidemiology of hematogenous osteomyelitis in Northwest China. To address this issue, we conducted a retrospective study using the data obtained from 259 patients with hematogenous osteomyelitis in a well-known hospital and conducted an epidemiological study. Statistical analysis of the epidemiological characteristics was conducted, and analyses of the microbiological characteristics, treatment methods and financial burden of hematogenous osteomyelitis in Northwest China were performed.

## 2 Method

### 2.1 Study design, setting, and data source

This study was conducted at Tangdu Hospital, Second Affiliated Hospital of Air Force Military Medical University, a tertiary medical center located in Xi’an, Shaanxi, Northwest China. Patient data were collected using the hospital’s electronic medical record information system. The key words “osteomyelitis” and “bone infection” were used for the search, and a time frame from January 1, 2010, to December 31, 2020, was used. Initially, the retrieved records were screened according to the inclusion and exclusion criteria, and for patients with multiple admissions, the first admission record was retained. Follow-up data were obtained through periodic patient review by searching the medical records and by telephone follow-up.

### 2.2 Inclusion and exclusion criteria

Inclusion criteria: Patients who met the criteria for a diagnosis of hematogenous osteomyelitis (divided into AHO and CHO according to the four-week disease course) (5); Patients with complete medical records and imaging and microbiological findings. Exclusion criteria: Patients with traumatic osteomyelitis; Patients with diabetic foot osteomyelitis; Patients with missing clinical data and incomplete medical records.

### 2.3 Observed indicators

Primary observed indicators: 1) Epidemiological status (Age and sex of onset, infection sites, Inflammatory markers, clinical symptoms and signs, history of fever and antibiotic use and etiology analysis); 2) Microbiology (Microorganism species analysis and drug susceptibility test); 3) Treatment methods (Conservative treatment and surgical treatment, surgical treatment usually divided into debridement, PMMA bone cements,calcium sulfate bone cements and calcium phosphate/calcium sulfate bone cements).

Secondary observed indicator: Financial burden (Pharmaceutical costs, diagnosis costs, anesthesia and surgery costs, materials costs, comprehensive care costs and blood and blood products costs).

### 2.4 Statistical analysis

Statistical analysis was performed with SPSS 26.0 software (SPSS Inc., Chicago, IL, USA). Data distributions were evaluated for normality using the Kolmogorov–Smirnov test. Continuous variables were expressed as the mean ± standard deviation (SD) or median with interquartile range (IQR) depending on the data distribution. While measurement data (general characteristics, laboratory markers and financial burden) were compared using the t test or rank-sum test. The Categorical variables (general characteristics, positive rates of inflammatory marker and clinical symptoms) were compared using Pearson’s chi-square test or Fisher’s exact test. Data was adjusted using logistic regression analysis. P values below 0.05 were considered significant.

## 3 Results

### 3.1 Baseline characteristics

#### 3.1.1 Sex and age (at first diagnosis)

This study included 172 males and 87 females, with a sex ratio of 1.98. The median age of the 259 patients was 33 years, and the AHO patients had a median age of 16 years; CHO patients had a median age of 41 years. The analysis revealed that there was a significant difference in the age of onset between the AHO and CHO patients (p<0.001), and the age of onset in the AHO patients was younger than that in the CHO patients. Among the 259 patients divided by age group, the top three age groups were 11-15 years old (36 patients), 6-10 years old (25 patients),and 61-65 years old (25 patients), 46-50 years old (24 patients). After being divided according to whether they had AHO or CHO, the age groups with the highest incidence were the 11-15 years old (22 patients) and 61-65 years old (22 patients) groups **Table 1**.

**Table 1:** Baseline data.

Items	HO	AHO	CHO	P value(AHO\CHO)
Number, *n*	259	96	163	
Male/female, *n*	172/87	64/32	108/55	0.946
Median age, *years*	33(14,52)	16(10,46]	41(22,56)	<0.001*
Adolescents/Adults, *n*	83/176	50/46	33/130	<0.001*
Single site/multiple sites, *n*	246/13	94/2	152/11	0.172
Extremities/Trunk sites, *n*	252/7	92/4	160/3	0.473
Median number of hospital days, *days*	22(13,29)	23(14,31)	21(13,29)	0.360
Re-admission antibiotic use/non-use, *n*	85/174	38/58	47/116	0.075
Number of people with history of fever/without fever, *n*	68/191	42/54	26/137	<0.001*
History of fever/No history of fever, *n*	30/229	17/79	13/150	0.018*
Median antibiotic duration since admission, *days*	15(8,22)	16(7,23)	15(9,21)	0.855

HO, hematogenous osteomyelitis; AHO, acute hematogenous osteomyelitis; CHO, chronic hematogenous osteomyelitis ; *P < 0.05.

#### 3.1.2 Infection sites

**Figure 1:**
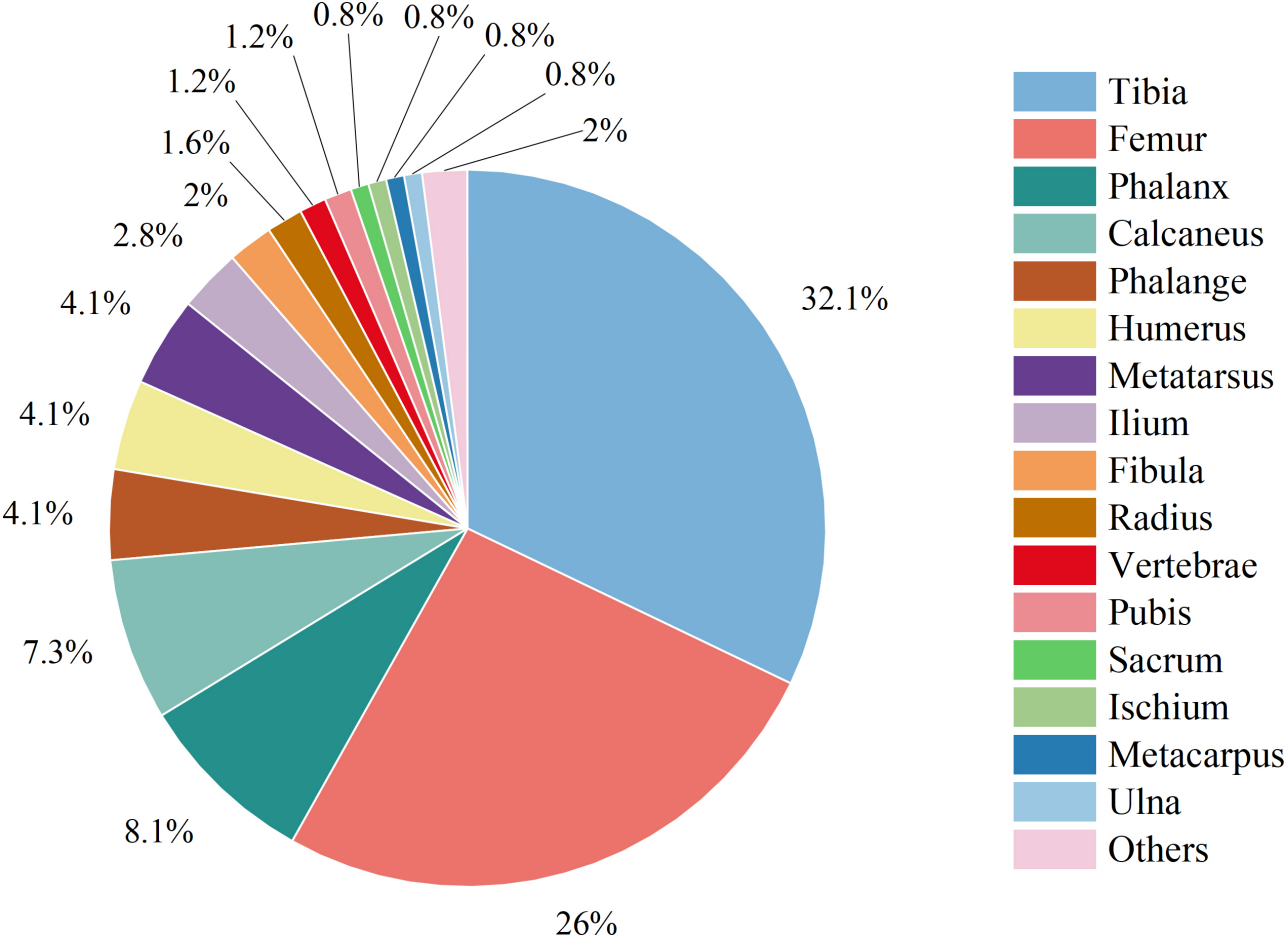
Distribution of single infection sites.

The total numbers of infections in the extremity and trunk sites were 252 (97.30%) and 7 (2.70%), respectively. Among all 259 infections, 246 (94.98%) were single infection, and 13 (5.02%) were multisite infections. The tibia (79 cases, 32.11%) was the most common site of solitary infection, followed by the femur (64 cases, 26.02%) and phalanges (20 cases, 8.13%) (**Figure 1**).

#### 3.1.3 History of fever and antibiotic use

Before admission, 68 patients had a history of fever, accounting for 26.25% of the total. Among them, 42 patients had fever before admission in the AHO group, accounting for 43.75% of the total number of patients with AHO, and 26 patients had fever before admission in the CHO group, accounting for 15.95% of the total number of patients with CHO. The difference was statistically significant (p=0.018). The number of febrile patients was significantly higher in the AHO group than in the CHO group. Before admission, 85 patients had a history of antibiotic use, accounting for 32.82% of the total, and most of these patients had taken cephalosporins (injection or oral administration).

#### 3.1.4 Etiology analysis

**Figure 2:**
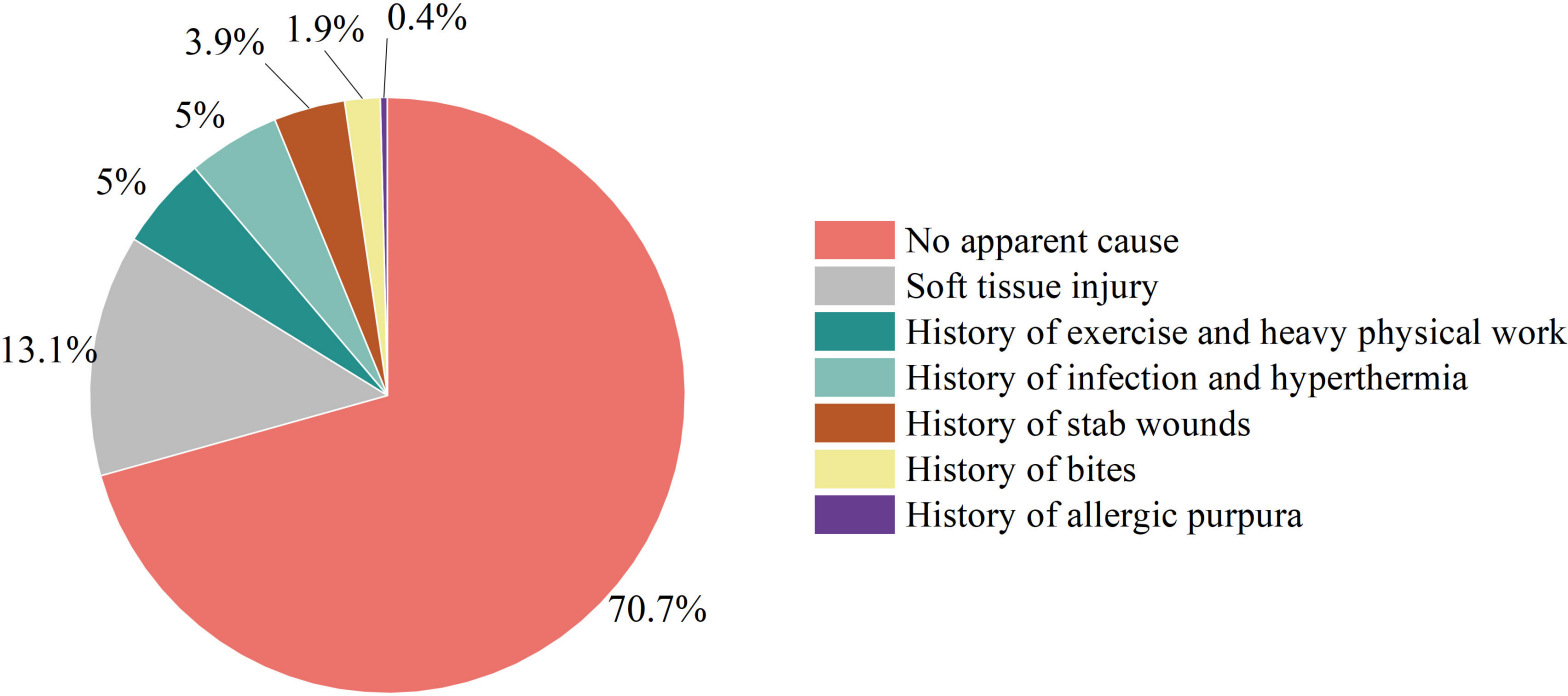
Etiology distribution chart.

Etiology analysis revealed that most cases of HO had no obvious cause (183/259, 70.66%); the number of cases caused by soft tissue injury (fall, abrasion, bruise, crush and crush) (34/259, 13.13%) followed the number of cases with no obvious cause. Exercise-induced illness (skipping, dancing, swimming, basketball sprain) and history of heavy physical labor (13/259, 5.02%) was the third most common cause. In addition, some patients exhibited a history of infection before onset (upper respiratory tract infection, chickenpox infection and skin infection); high fever (13/259, 5.02%); a history of being stabbed, punctured or injected (10/259, 3.86%); bite history (5/259, 1.93%); and a history of allergic purpura (1/259, 0.39%)(**Figure 2**).

### 3.2 Inflammatory markers and clinical symptoms and signs

As shown in **Table 2**, among the preoperative (upon admission) serum inflammatory markers, the rate of ESR positivity was the highest at 67.58% (148/219), followed by hs-CRP at 66.67% (132/198), and the rate of WBC positivity was at 24.90% (63/253). According to the AHO and CHO classifications, the rates of positivity for WBC were 38.30% and 16.98%, the rates of positivity for hs-CRP were 69.33% and 65.04%, and the rates of positivity for ESR were 75.61% and 62.77%, respectively. There were significant differences between the AHO and CHO patients in the WBC levels, and the AHO patients had a higher WBC count than the CHO patients (p<0.001). The baseline age of the AHO patients was significantly younger than that of the CHO patients (p<0.001). To reduce the age-induced bias, we performed age-adjusted analysis. After adjusted, positive rate of ESR showed significant difference (p = 0.014).

**Table 2:** Inflammatory markers and clinical symptoms.

Items	HO	AHO	CHO	P value	Adjusted P	OR(95%CI)
Serum levels of inflammatory markers						
WBC (×10^9^/L)	6.70(5.28, 9.76)	7.90(5.91, 12.23)	6.23(5.09, 7.87)	<0.001^*^	0.001^*^	1.13(1.05 to 1.21)
Hs-CRP(mg/L)	7.19(2.69, 40.03)	12.30(3.07, 60.80)	5.78(1.85, 29.51)	0.123	0.068	1.01(1.00 to 1.01)
ESR (mm/h)	28(12, 68)	37.50(16, 69.50)	25(10, 66.50)	0.210	0.429	1.00(0.99 to 1.01)
Positive rates of inflammatory markers						
WBC, %(+/n)	24.90%(63/253)	38.30%(36/94)	16.98%(27/159)	<0.001^*^	<0.001^*^	3.13(1.69 to 5.82)
hs-CRP, %(+/n)	66.67%(132/198)	69.33%(52/75)	65.04%(80/123)	0.534	0.185	1.58(0.80 to 3.13)
ESR, %(+/n)	67.58%(148/219)	75.61%(62/82)	62.77%(86/137)	0.050	0.014^*^	2.28(1.18 to 4.39)
Positive rates of clinical symptoms						
Redness of the skin, *%(+/n)*	37.45%(97/259)	46.88%(45/96)	31.90%(52/163)	0.016^*^	0.014*	1.98(1.15 to 3.42)
Swelling, *%(+/n)*	67.57%(175/259)	76.04%(73/96)	62.58%(102/163)	0.025^*^	0.066	1.74(0.96 to 3.12)
Fever, *%(+/n)*	43.24%(112/259)	60.42%(58/96)	33.13%(54/163)	<0.001^*^	<0.001^*^	2.82(1.64 to 4.86)
Pain, *%(+/n)*	85.71%(222/259)	89.58%(86/96)	83.44%(136/163)	0.172	0.359	1.46(0.65 to 3.26)
Dysfunction, *%(+/n)*	16.99%(44/259)	23.96%(23/96)	12.88%(21/163)	0.022^*^	0.012^*^	2.46(1.22 to 4.95)

WBC, white blood cell; Hs-CRP, Hypersensitive- C reactive protein; ESR, erythrocyte sedimentation rate; HO, hematogenous osteomyelitis; AHO, acute hematogenous osteomyelitis; CHO, chronic hematogenous osteomyelitis; OR, odds ratio; CI, confidence intervals; ^*^, P<0.05.

The manifestations of inflammation were divided into redness of the skin, swelling, fever, pain and dysfunction according to the consultation and physical examination upon admission. Pain affected the highest number of patients at 222, which was followed by 175 patients with swelling and 44 patients with functional impairment. There were significant differences in the incidence of redness of the skin, swelling, fever, and dysfunction between the AHO and CHO patients (p<0.05), and these clinical symptoms were more common in the AHO patients than in the CHO patients. The baseline age of the AHO patients was significantly younger than that of the CHO patients (p<0.001). To reduce the age-induced bias, we performed age-adjusted analysis. After adjusted, positive rate of swelling symptom showed no significant difference (p = 0.066).

### 3.3 Microorganism species analysis and drug susceptibility test

In our study, a total of 171 patients had documented pathogen cultures. The rate of positivity among all cultures was 71.93% (123 cases), of which 78.86% (97 cases) involved single microorganism infections. Among those with single microorganism infections, Gram-positive bacteria accounted for 86.60% (84 cases), Gram-negative bacteria accounted for 13.40% (13 cases), and SA accounted for 63.92% (62 cases), including MSSA 56.70% (55 cases) and MRSA 7.22% (7 cases). Among the polymicrobial infections, 46.15% (12 cases) involved only Gram-positive bacteria, 11.54% (3 cases) involved only Gram-negative bacteria, and the rest were mixed bacterial infections.

As shown in **Table 3**, among the 202 strains of pathogenic microorganisms detected, 160 strains were Gram-positive bacteria, 41 strains were Gram-negative bacteria and 1 strain was fungi. The top three were MSSA with 100 strains (49.50%), MRSA with 13 strains (6.44%), and MRSE with 9 strains (4.46%). The top three pathogenic bacteria in the AHO group were 53 strains of MSSA, 8 strains of MRSA and 3 strains of MRSE. SA accounted for 75.61% (31/41) of the single microorganism infections in AHO patients. The top three pathogenic bacteria cultured in the CHO patients were 47 strains of MSSA, 6 strains of MRSE and 6 strains of Enterobacter cloacae. SA accounted for 55.36% (31/56) of the single microorganism infections in the CHO patients.

**Table 3:** Microorganism species and quantities.

Microorganism species	Strains	Gram stain
MSSA	100	Gram positive
MRSA	13	Gram positive
MRSE	9	Gram positive
Corynebacterium	8	Gram positive
Enterococcus faecium	4	Gram positive
Enterococcus faecalis	3	Gram positive
Staphylococcus hemolyticus	3	Gram positive
Staphylococcus hominis	3	Gram positive
Staphylococcus epidermidis	2	Gram positive
Streptococcus hemolyticus	3	Gram positive
Staphylococcus suis	3	Gram positive
Bacillus subtilis	2	Gram positive
Streptococcus dysgalactiae	1	Gram positive
Streptococcus constellatus	1	Gram positive
Hemolytic gemini	1	Gram positive
Enterococcus avis	1	Gram positive
Staphylococcus mimicus	1	Gram positive
Staphylococcus lugdunensis	1	Gram positive
Staphylococcus schneideri	1	Gram positive
Enterobacter cloacae	7	Gram negative
Pseudomonas aeruginosa	7	Gram negative
Escherichia coli	5	Gram negative
Proteus mirabilis	5	Gram negative
Klebsiella pneumoniae	4	Gram negative
Proteus vulgaris	4	Gram negative
Morganella morganii	2	Gram negative
Acinetobacter baumannii	2	Gram negative
Flavobacterium breve	1	Gram negative
Bacteroides	1	Gram negative
Leclercia adcarboxglata	1	Gram negative
Aeromonas hydrophila	1	Gram negative
Aggregatibacter aphrophilus	1	Gram negative
Candida parapsilosis	1	Fungi

MSSA, Methicillin sensitive Staphylococcus aureus.

MRSA, Methicillin resistant Staphylococcus aureus.

MRSE, Methicillin resistant Staphylococcus epidermidis.

Through the analysis of drug susceptibility results it was observed that, among the Gram-positive bacteria (**Table 4**), MSSA was most sensitive to linezolid (99%), followed by vancomycin (98%), oxacillin (98%) and rifampicin (98%). The MRSA strains were most sensitive to linezolid (100%), vancomycin (100%), and rifampicin (100%). The MRSE strains were most sensitive to linezolid (100%) and vancomycin (100%). Regarding Gram-negative bacteria (**Table 5**), E. cloacae was most sensitive to aztreonam (100%) and piperacillin/tazobactam sodium (100%). Escherichia coli was most sensitive to piperacillin/tazobactam sodium (100%). Pseudomonas aeruginosa was most sensitive to ciprofloxacin (85.71%) and imipenem (85.71%).

**Table 4:** Antimicrobial resistance of the main Gram-positive bacteria.

Antimicrobial	MSSA(n=100)		MRSA(n=13)		MRSE(n=9)	
	strains	S(%)	strains	S(%)	strains	S(%)
AMC	85	85	0	0	0	0
SAM	66	66	0	0	0	0
OXA	98	98	0	0	0	0
DAP	68	68	8	61.54	6	66.67
ERY	43	43	1	7.69	0	0
CIP	94	94	12	92.31	3	33.33
SXT	93	93	12	92.31	4	44.44
QDA	56	56	9	69.23	1	11.11
RIF	98	98	13	100	8	88.89
LNZ	99	99	13	100	9	100
CLI	41	41	1	7.69	0	0
MXF	68	68	8	61.54	5	55.56
PEN	6	6	0	0	0	0
GEN	88	88	12	92.31	3	33.33
TCY	84	84	11	84.62	5	55.56
CRO	67	67	0	0	0	0
CZO	20	20	–	–	0	0
VAN	98	98	13	100	9	100
LEV	84	84	7	53.85	4	44.44

MSSA, Methicillin sensitive Staphylococcus aureus; MRSA, Methicillin resistant Staphylococcus aureus; MRSE, Methicillin resistant Staphylococcus epidermidis.

**Table 5:** Antimicrobial resistance of the main Gram-negative bacteria.

Antimicrobial	E.cloacae(n=7)		P. aeruginosa (n=7)		E. coli(n=5)	
	strains	S(%)	strains	S(%)	strains	S(%)
AMK	6	85.71	5	71.43	4	80
AMC	0	0	0	0	2	40
ATM	7	100	5	71.43	0	0
ETP	6	85.71	0	0	2	40
TZP	7	100	5	71.43	5	100
CIP	5	71.43	6	85.71	1	20
SXT	3	42.86	0	0	0	0
MEM	5	71.43	5	71.43	4	80
GEN	5	71.43	2	28.57	1	20
TCC	1	14.29	1	14.29	1	20
FEP	5	71.43	3	42.86	0	0
CRO	3	42.86	0	0	0	0
CTX	3	42.86	0	0	0	0
CAZ	3	42.86	4	57.14	1	20
CZO	0	0	0	0	0	0
TOB	5	71.43	0	0	0	0
IPM	6	85.71	6	85.71	4	80
LEV	5	71.43	4	57.14	1	20

### 3.4 Treatment strategy

The treatment of hematogenous osteomyelitis usually includes conservative treatment and surgical treatment. Surgical treatment is usually divided into debridement (including simple debridement, debridement plus drip drainage, and debridement plus VSD suction), bone cement (nonabsorbable bone cements, such as PMMA; absorbable bone cement, such as calcium sulfate (CS); and calcium phosphate/calcium sulfate (CS/CP)) and amputation in some patients with severe infections (**Table 6**).

**Table 6:** Treatment strategy and remission rate.

Treatment strategy	Patients	Constituent ratio/%	Remission rate/%
Conservative treatment	75	28.96	90%(54/60)
Debridement	126	48.65	88.18%(97/110)
Debridement and PMMA bone cement	17	6.56	88.24%(15/17)
Debridement and CS、CS/CP bone cement	23	8.88	100%(23/23)
Amputation	18	6.95	–

PMMA, polymethyl methacrylate; CS, calcium sulfate; CS/CP, calcium sulfate/calcium phosphate.

Regarding the antibiotics given on admission, 235 patients (90.73%) received intravenous antibiotics, and 24 patients (9.27%) received oral antibiotics. Regarding intravenous antibiotic therapy, 96 patients (40.85%) received single antibiotic therapy, of which cefazolin (25 patients,26.04%) was the most commonly used antibiotic.

All patients were followed up for at least 12 months; 31 patients (11.97%) were lost to follow-up, and 18 patients (6.95%) had amputations in the hospital. A total of 189 patients(90%) were in remission, and 21 patients (10%) were in relapse. Among them, after conservative treatment, the remission rate was 90%, the remission rate of the patients with one-stage debridement was 88.18%, the remission rate of the patients with one-stage debridement plus antibiotic-loaded PMMA bone cement exclusion was 88.24%, and the patients who had CS or CS/CP had a 100% remission rate.

**Figure 3:**
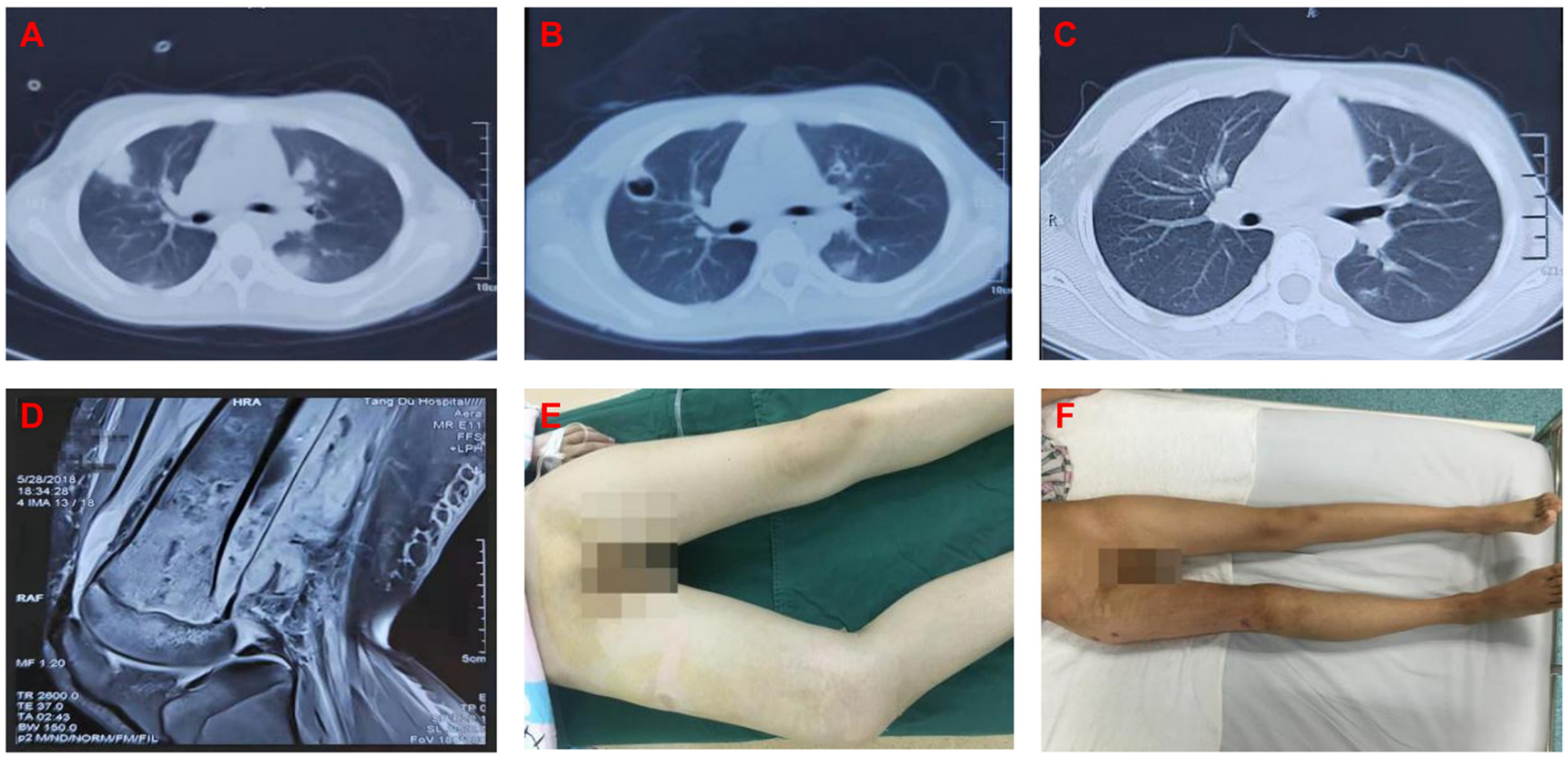
**(A)** 2018.05.20 lung CT(Before surgery): It was indicated that there were multiple abscesses in the lung. **(B)** 2018.05.28 lung CT(Before surgery): It was indicated that the lung abscesses shrinked after treatment. **(C)** 2018.06.20 lung CT(After surgery): It was indicated that the lung abscesses almost disappeared. **(D)** 2018.05.28 MRI(Before surgery): It was indicated that there were edemas in the medullary cavity of the metaphysis soft tissue and surrounded the distal femur. **(E)** General photo at admission: It was indicated that forced posture caused by the disease and limb swelling. **(F)** General photo at discharge: It was indicated that the right leg of the patient was free of pain and swelling, the hip and knee joints could move freely as well.

Treatment of severe patients with sepsis and pulmonary infection. 15 of our patients (5.80%) had coexisting pulmonary infections. For patients with pulmonary infection, the precise diagnosis should be made first to avoid a missed diagnosis or a misdiagnosis. In addition to surgical treatment of local infections of the limbs, systemic intravenous therapy should be combined with appropriate antibiotics. In this subset of patients, we found that oxacillin was very effective unlike other antibiotics. All patients were successfully treated, and the lung infection was cured,such as case 1(**Figure 3**).

### 3.5 Financial burden

The total Financial burden for the hospitalized patients with hematogenous osteomyelitis was 8,832,910 RMB. The median total cost for the hospitalized patients with hematogenous osteomyelitis was 25,754 RMB (11,502 RMB, 47,661 RMB). Among the main costs of the patients with hematogenous osteomyelitis, pharmaceutical costs accounted for the largest proportion (36.17%), followed by material costs (24.06%), anesthesia and surgery costs (17.47%), diagnosis costs (16.10%), comprehensive care costs (5.02%) and blood product costs (1.17%)(**Table 7**).

**Table 7:** Financial burden and financial burden classification comparison.

Groups		Total	Pharmaceuticals	Diagnosis	Anesthesia and Surgery	Materials	Comprehensive care	Blood and Blood products
Financial burden	Per patient	25754(11502,47661)	10084(3816,17129)	4220(2573,6608)	4547(2264,8106)	2630(1077,15473)	1230(730,1862)	832(516,1184)
	Total Financial burden	8832910	3194838	1422360	1543212	2125337	443382	103780
	Percentage	100%	36.17%	16.10%	17.47%	24.06%	5.02%	1.17%
Acute/Chronic	AHO	23682(10335,48964)	9458(3240,17298)	4167(2527,7895)	4913(2109,8434)	2048(1062,10972)	1293(757,1892)	654(516,1350)
	CHO	26890(12835,47053)	10309(4607,16690)	4261(2633,6359)	4480(2264,8035)	3259(1108,18515)	1149(715,1862)	832(516,1078)
	P values	>0.05	>0.05	>0.05	>0.05	>0.05	>0.05	>0.05
Sex	Male	27592(13438,51180)	10633(4837,20477)	4516(2754,7288)	5289(2624,8581)	3020(1190,15536)	1280(804,1947)	643(516,1116)
	Female	17928(8819,38284)	7504(3152,13909)	3506(2497,6359)	3876(1768,6228)	1920(905,12220)	1003(570,1618)	952(432,1370)
	P values	<0.05	<0.05	>0.05	<0.05	>0.05	<0.05	>0.05
Age range	0-17	20486(10402,44453)	5841(2797,13241)	4067(2577,5976)	4557(2432,7929)	2163(1020,11693)	1185(754,1593)	524(436,1372)
	18-59	31169(14451,52405)	11631(7144,22303)	4298(2519,7069)	5165(2277,8546)	3180(1119,17964)	1320(692,2089)	992(516,1262)
	≥60	21303(10576,42746)	8611(3719,13707)	4147(2692,6575)	4171(1788,8035)	2389(1185,13618)	1072(720,1605)	570(516,1072)
	P values	>0.05	<0.05	>0.05	>0.05	>0.05	>0.05	>0.05
Top 3 infection sites	Tibia	24060(12917,47305)	9127(4306,16620)	3930(2554,5389)	4369(2369,8824)	3025(1216,18711)	1019(715,1483)	619(524,1192)
	Femur	33360(21041,57028)	12608(7744,22544)	5818(3664,8751)	6036(3229,9798)	3142(1258,14991)	1543(1054,2129)	1032(516,1500)
	Phalanx	10950(7007,17995)	5775(2246,9198)	2104(1554,3272)	2440(1788,3482)	930(378,3945)	703(499,1268)	-
	P values	<0.05	<0.05	<0.05	<0.05	<0.05	<0.05	>0.05
Conserva tive/ Surgical	Conservative	8035(4168,14460)	2928(886,5658)	2779(1762,4619)	1145(413,2066)	424(192,1416)	720(304,1290)	892(607,1569)
	Surgical	35838(20942,55853)	11496(7841,20211)	4832(3316,7550)	6100(4048,9638)	5057(1665,18870)	1410(909,2085)	654(516,1128)
	P values	<0.05	<0.05	<0.05	<0.05	<0.05	<0.05	>0.05

AHO, acute hematogenous osteomyelitis; CHO, chronic hematogenous osteomyelitis

According to the analysis of the financial burden based on disease classification, there was no significant difference between acute osteomyelitis and chronic osteomyelitis patients in the total hospitalization expenses or other various expenses. There were significant differences (p<0.05) in the pharmaceutical costs, comprehensive care costs and total personal costs between the male and female patients, and the male patients were associated with higher costs than the female patients. According to the different age groups, after the patients were divided into age groups of 0-17 years old, 18-59 years old and 60 years old and older, it was observed that there were significant differences in the pharmaceutical costs (p<0.05) among the three age groups, with the patients aged 18-59 being associated with the highest, followed by the patients aged 60 and older and the patients aged 0-17. Regarding the infection site, the three single infection sites with the highest incidence were the tibia, femur, and phalanges, and significant differences were observed among the three sites in terms of the associated total individual costs, pharmaceutical costs, diagnosis costs, anesthesia and surgery costs, material costs, and comprehensive care costs (p<0.05). Femur osteomyelitis was associated with the highest costs, followed by tibia and phalanges osteomyelitis. The treatment methods were mainly divided into conservative treatment and surgical treatment. There were significant differences in the total hospitalization costs, pharmaceutical costs, diagnosis costs, anesthesia and surgery costs, materials costs and comprehensive care costs (p<0.05). The costs of surgical treatment was significantly higher than the costs of conservative treatment.

## 4 Discussion

To assess etiology, this study involved an epidemiological analysis of 259 Chinese HO patients and revealed that most cases of HO had no obvious cause; the number of cases with no obvious cause was followed by the number of cases due to soft tissue injuries (falls, abrasions, bruises, bruises, and crushes), and histories of sports involvement (sprains from skipping rope, dancing, swimming, playing basketball), heavy physical labor, infection (upper respiratory tract infection, chickenpox infection and skin infection), high fever, stabbing injury, puncture injury and injection were observed before disease onset. Some patients also had histories of bite injuries, as well as of anaphylactoid purpura, which have been reported in studies (17–20).

Age of onset: Regarding the patient ages, the median age of the 259 HO patients was 33 years old; the median age of the AHO patients was 16 years old and the median age of the CHO patients was 41 years old. The analysis revealed that the age of the AHO patients was significantly younger than that of the CHO patients (p<0.001). AHO most often occurs in adolescent patients, and this may be related to a weakened immune system or underdevelopment (21, 22).

Infection site: Regarding the infection sites, in our study, the most common single infection sites were the tibia (79 cases), femur (64 cases) and phalanges (20 cases), among which the most common site of AHO was the tibia (37 cases), and the most common site of CHO was the femur (44 cases). AHO occurs in the tibia and femur of children and adolescents (2). This finding is consistent with the results of many domestic and foreign studies (9, 14, 15). The anatomical location of AHO in children is the metaphysis, which may be related to the abundant but slow blood flow in this part of the bone (21, 23).

Inflammatory markers: Regarding serum inflammatory markers, Kenneth L. Urish (24) et al. believed that the elevated levels of ESR and CRP were not specific for osteomyelitis. Although these serum inflammatory markers are not specific, they can be used for the monitoring and follow-up of patients (25). CRP has been shown to be a very good and inexpensive marker for follow-up (26, 27). Our study showed that ESR was the highest rate of positivity and WBC was the lowest. CRP levels can more accurately reflect the changes in infection, and the rate of the decline in the ESR is significantly slower than that in CRP levels. Therefore, CRP plays a beneficial role in monitoring the condition (2, 27). To reduce the age-induced bias, we performed age-adjusted analysis, there were significant differences in the rate of positivity of WBC and ESR between the AHO and CHO patients (p<0.05). Our study showed that acute osteomyelitis is more sensitive to serological changes, while chronic osteomyelitis is less sensitive to serological changes. In addition, studies have reported that the ESR can reflect the formation of abscesses (28, 29).

Stucken (30) et al. reported that approximately 20% of patients were still infected even when the WBCs, ESR and CRP levels were normal. In our study, 15.83% (41/259) of the patients had normal WBC levels, ESR and hs-CRP levels; among them, AHO accounted for 31.71% (13/41), and CHO accounted for 68.29% (28/41). The clinical diagnosis needs to include the patient’s bone biopsy results, microbial culture results, clinical symptoms and signs, and imaging data. The preferred diagnostic criterion for osteomyelitis is a positive bacterial culture on a bone biopsy, although clinical manifestations, laboratory tests, and imaging data can also aid in the diagnosis (31). Regarding the clinical manifestations, the common inflammatory manifestations of osteomyelitis include redness, swelling, heat, pain and dysfunction. Osteomyelitis should be considered at the time of diagnosis in patients with musculoskeletal pain and systemic symptoms (32).

Microorganism characteristics: Regarding the rate of positivity for pathogenic bacterial culture, that observed in our study was 71.93%. The rate of positivity for pathogenic bacteria in AHO patients was 50/67 (74.63%), and that in CHO patients was 73/104 (70.19%). The top three strains of pathogenic bacteria culture were MSSA (100 strains), MMSA (13 strains) and MRSA (9 strains), and the pathogen with the most single microorganism infection cases was SA (62/97, 63.92%), including MSSA (55/97, 56.70%)and MRSA (7/97, 7.22%). SA accounted for 75.61% (31/41) of the single microorganism infections in AHO patients, and SA accounted for 55.36% (31/56) of the single microorganism infections in CHO patients. Wang et al. (15) found that the incidence of hematogenous osteomyelitis, mainly caused by Staphylococcus aureus, is still high in Southwestern China, and SA accounted for 76.2% of the single-microbial infections in CHO patients. Staphylococcus aureus is the most common pathogen of bone infections and is very destructive (33). Understanding the distribution and sensitivity of pathogenic bacteria in blood-borne osteomyelitis in recent years in this region is of great importance for guiding clinical medication. It takes 1-2 weeks to culture microorganisms and perform drug susceptibility tests. To relieve symptoms as soon as possible, a broad-spectrum antibacterial drug with high sensitivity can be empirically selected before the bacterial culture results are revealed, and then antibacterial drugs can be selected after the bacterial culture and drug susceptibility results are acquired.

Antibiotic strategy: Treatment of hematogenous osteomyelitis usually includes surgical and nonsurgical treatment. Nonsurgical treatment is mainly based on early, adequate, and full courses of antibiotics, which mainly involves intravenous antibiotics.

Antibiotic selection is mainly based on the results of drug susceptibility tests and the minimum inhibitory concentration and PK of pathogenic bacteria. At the same time, the safety characteristics of antibiotics, such as bone permeability PD and liver and kidney metabolism, need to be considered. It has been reported that the combined use of antibiotics is recommended in cases of multiple pathogenic bacterial infections (34).

MSSA infection accounted for a large proportion of our cases. Regarding the empirical choice of first-line antibiotics for MSSA infection, there are treatment guidelines indicating that oxacillin/nafcillin or cefazolin is recommended in areas where the incidence of MRSA osteomyelitis is less than 10%. When the incidence of MRSA osteomyelitis is higher than 10%, the use of clindamycin or vancomycin is recommended (5). Some treatment guidelines recommend first-generation cephalosporins and clindamycin (35). Regarding this controversial issue, our research group found that for MSSA, oxacillin had a good effect (the sensitivity rate was 98%). However, according to the drug susceptibility results, the rate of MSSA sensitivity to oxacillin was only lower than that to high-grade linezolid, which is the same as that to the antibiotic vancomycin (the sensitivity rate is 98%), indicating that it has good PK. Additionally, oxacillin has good permeability and bone tissue, and the MIC value remains stable when the ph value changes (36, 37).

However, MSSA is also highly sensitive to vancomycin. In our study, the sensitivity of MSSA was as high as 98%, but the use of this drug may lead to the recurrence of infection (38). We had 1 patient(case 1) with an MSSA infection, as mentioned above, and vancomycin was effective. However, the patient relapsed after 7 days, and we adjusted the use of oxacillin in time so that the patient’s condition improved. Therefore, our study may provide evidence for the use of oxacillin instead of vancomycin as a first-line drug for MSSA blood-borne osteomyelitis.

The recommended first-line drugs for the antibiotic treatment of MSSA are cefazolin and oxacillin (5, 39). In our treatment of patients with MSSA infection, cefazolin was used as the initial antibiotic more, but we found that cefazolin did not work well for some patients, and after changing to oxacillin, the effect was more obvious. The reasons may be that the MIC value of oxacillin MIC is usually 0.25 in our hospital, which is lower than that of cefazolin (MIC=2); oxacillin is less used in this area than cefazolin, so the drug resistance is poor; cefazolin itself has a cefazolin inoculum effect (CIE) (40–42); and the use of oxacillin in this study resulted in no serious toxic effects.

Multiple site infection: In our study, approximately 5.80% of the patients with blood-borne bone infections exhibited complications with pulmonary infections. High fever usually persists or recurs in critically ill patients with pulmonary infection and sepsis. In the case of a clear diagnosis of hematogenous osteomyelitis, if a high fever is observed during treatment, it is necessary to suspect infection in other parts of the body. It is recommended that lung CT and other examinations are performed in time to avoid missed diagnoses. The question of whether hematogenous osteomyelitis or pulmonary infection is the primary disease needs to be clarified.

Surgical intervention: Regarding conservative versus surgical treatment, the general indications for surgical intervention are currently considered to include the presence of subperiosteal or soft tissue abscesses, sequestrum formation, sinus formation, and surrounding soft tissue involvement (24). However, in response to this problem, we believe that the timing of surgical intervention is very important, and the time of surgical intervention for acute hematogenous osteomyelitis may directly determine the prognosis of patients. When acute hematogenous osteomyelitis forms an abscess around the bone tissue, there is a high chance that it will progress into chronic osteomyelitis, even if debridement is performed at this time. Therefore, surgery at the early stage of abscess formation can inhibit the spread of infection, improve the prognosis of patients and improve patient outcomes. In patients with chronic hematogenous osteomyelitis, a sequestrum has already formed or there is a sinus tract, and active surgical treatment should be performed to remove the necrotic tissue, improve the blood supply to the bone tissue, eliminate dead space and provide stability.

The traditional methods of surgical intervention are mainly debridement. During debridement, VSD suction, drip drainage and soft tissue coverage are combined. However, there is a problem with dead space after debridement, which may become a potential risk factor for infection recurrence. Therefore, some scholars proposed that when there is dead space formation, an auxiliary removal technique can be used to eliminate the dead space, and the materials for removal mainly include nonabsorbable PMMA bone cement (43, 44), absorbable calcium sulfate bone cement (45–48), calcium sulfate/calcium phosphate bone cement (49) and so on.

For the exclusion material, both nonabsorbable PMMA and absorbable CS bone cement can deliver high doses of antibiotics to the surrounding bone tissue and have the advantage of managing the dead space (34). CS bone cement can release high concentrations of antibiotics faster than PMMA and has the advantages of being biodegradable and not requiring secondary surgical removal (50). In this study, debridement combined with CS cement and CS/CP cement achieved a 100% remission rate in 23 patients. It was better than traditional debridement combined with drip drainage (88.18%) and PMMA exclusion (88.24%), but the result was not statistically significant due to the small number of cases. The advantage of a CS or CS/CP may be that it has better sustained release of antibiotics and resorption and osteogenic effects.

Our research group has previously confirmed that a CS/CP has a better effect than CS in the treatment of chronic osteomyelitis, the infection recurrence rate is low, and this approach can be used as a good choice for the treatment of bone infection. Both fillers have the advantage of being biodegradable. The difference is mainly in the absorption rate during the early stage. The absorption rate of CS is too fast; however, the absorption rate of CS/CP is almost the same as the rate of osteogenesis, leaving no new cavities (51). It is worth conducting in-depth basic and clinical research.

Regarding the financial burden, the median total costs per patient was 25,754 RMB, and the average treatment costs for 259 patients was 34,104 RMB. Among the main costs, drugs accounted for the largest proportion, accounting for 36.17%. Jiang (13) and others analyzed the financial burden of treating traumatic osteomyelitis patients in Southwestern China. After exchange rate conversion and calculation, the median total costs per patient was 73,528 RMB, and the average treatment costs for 278 patients was 88,751 RMB. Among the costs, materials accounted for the largest proportion, at 61%. Obviously, the financial burden of treating traumatic osteomyelitis is higher than that of treating hematogenous osteomyelitis. The reason may be that the treatment of posttraumatic osteomyelitis often requires the placement of internal and external fixators *via* surgery, which increases the expenditure for materials and requires longer treatment cycles. We only analyzed the medical expenses during the patient’s first hospitalization and did not analyze the patient’s multiple hospitalization expenses and other expenses, such as out-of-hospital expenses (examination, testing, and treatment in other hospitals). Therefore, the expenses of HO inpatients may have been underestimated.

## 5 Conclusion

In our study of 259 patients with hematogenous osteomyelitis, males had a higher incidence, and there was a male-to-female ratio of 1.98. Hematogenous osteomyelitis occurs in the lower extremities, with the three most common individual sites of infection being the tibia, femur, and phalanges. The rate of ESR positivity was the highest in the preoperative laboratory examination. The most common pathogen associated with HO infection was MSSA. Oxacillin has good PK and PD and is recommended as the first-line drug. Some blood-borne bone infections may lead to complications, such as pulmonary infection through bacteremia, which requires early detection to avoid a missed diagnosis. Regarding surgical intervention, debridement plus absorbable calcium sulfate bone cement and calcium sulfate calcium phosphate bone cement exclusion have achieved good therapeutic effects, but they are worthy of further in-depth research. Regarding the financial burden, the median total costs per patient was 25,754 RMB. The financial burden of blood-borne osteomyelitis was lower than that of traumatic osteomyelitis. Among the main costs, drugs accounted for the largest proportion.

## Data availability statement

The original contributions presented in the study are included in the article/supplementary material. Further inquiries can be directed to the corresponding authors.

## Ethics statement

The studies involving human participants were reviewed and approved by Medical Ethics Committee of the Second affiliated hospital, Air Force Medical University. Written informed consent to participate in this study was provided by the participants’ legal guardian/next of kin. Written informed consent was obtained from the individual(s) for the publication of any identifiable images or data included in this article.

## Author contributions

SL contributed to data collection and writing the paper and performed surgeries. LW and WL contributed to data collection and data analysis, performed surgeries, and wrote the paper. GW and ZZ contributed to data collection. HG and CF contributed to data collection. SL and JR contributed to patient follow-up. YoZ. and YuZ contributed to overall planning and data analysis and performed surgeries. All authors contributed to the article and approved the submitted version.
